# High Sensitivity of Late Gadolinium Enhancement for Predicting Microscopic Myocardial Scarring in Biopsied Specimens in Hypertrophic Cardiomyopathy

**DOI:** 10.1371/journal.pone.0101465

**Published:** 2014-07-07

**Authors:** Tetsuo Konno, Kenshi Hayashi, Noboru Fujino, Yoji Nagata, Akihiko Hodatsu, Eiichi Masuta, Kenji Sakata, Hiroyuki Nakamura, Masa-aki Kawashiri, Masakazu Yamagishi

**Affiliations:** 1 Division of Cardiovascular Medicine, Kanazawa University Graduate School of Medicine, Kanazawa, Japan; 2 Research and Education Center for Innovative and Preventive Medicine, Kanazawa University, Kanazawa, Japan; 3 Department of Public Health, Graduate School of Medical Science, Kanazawa University, Kanazawa, Japan; Tokai University, Japan

## Abstract

**Background:**

Myocardial scarring can be assessed by cardiac magnetic resonance imaging with late gadolinium enhancement and by endomyocardial biopsy. However, accuracy of late gadolinium enhancement for predicting microscopic myocardial scarring in biopsied specimens remains unknown in hypertrophic cardiomyopathy. We investigated whether late gadolinium enhancement in the whole heart reflects microscopic myocardial scarring in the small biopsied specimens in hypertrophic cardiomyopathy.

**Methods and Results:**

Twenty-one consecutive patients with hypertrophic cardiomyopathy who were examined both by cardiac magnetic resonance imaging and by endomyocardial biopsy were retrospectively studied. The right interventricular septum was the target site for endomyocardial biopsy in all patients. Late gadolinium enhancement in the ventricular septum had an excellent sensitivity (100%) with a low specificity (40%) for predicting microscopic myocardial scarring in biopsied specimens. The sensitivity of late gadolinium enhancement in the whole heart remained 100% with a specificity of 27% for predicting microscopic myocardial scarring in biopsied specimens. Quantitative assessments of fibrosis revealed that the extent of late gadolinium enhancement in the whole heart was the only independent variable related to the microscopic collagen fraction in biopsied specimens (β  =  0.59, 95% confident interval: 0.15 – 1.0, p  =  0.012).

**Conclusions:**

Although there was a compromise in the specificity, the sensitivity of late gadolinium enhancement was excellent for prediction of microscopic myocardial scarring in hypertrophic cardiomyopathy. Moreover, the severity of late gadolinium enhancement was independently associated with the quantitative collagen fraction in biopsied specimens in hypertrophic cardiomyopathy. These findings indicate that late gadolinium enhancement can reflect both the presence and the extent of microscopic myocardial scarring in the small biopsied specimens in hypertrophic cardiomyopathy.

## Introduction

Hypertrophic cardiomyopathy (HCM) is a primary cardiac disorder characterized by myocardial hypertrophy in the absence of other loading conditions, such as aortic stenosis or hypertension [1]. Histopathological criteria for HCM include myocyte hypertrophy, myocyte disarray, nuclear hypertrophy, interstitial fibrosis and myocardial scarring [2], which can be directly assessed by endomyocardial biopsy [3], [4]. However, potential pitfalls of endomyocardial biopsy include sampling error [5]–[8]. Recent data demonstrated that the sensitivity of endomyocardial biopsy for diagnosing HCM is as high as 75% [2].

Myocardial scarring is defined as increased collagen with evidence of cardiomyocyte loss [9]. In HCM patients, myocardial scarring is patchy and often found in the ventricular septum [9]–[12]. Determination of myocardial scarring is clinically important because myocardial scarring is associated with unfavorable clinical manifestations such as fatal ventricular arrhythmias and the development of heart failure in HCM [13]–[16]. Cardiac magnetic resonance imaging (CMR) with late gadolinium enhancement (LGE) imaging has been established recently as the imaging method for the assessment of myocardial scarring [5], [13]–[16]. The development of LGE-CMR techniques enables us to examine diagnostic accuracy of LGE-CMR for determination of microscopic myocardial scarring in biopsied specimens in HCM. The aim of this study was to investigate whether LGE-CMR can be used to predict myocardial scarring in small biopsied specimens in HCM using histopathological findings as the reference standard for fibrosis.

## Methods

### Study population

Twenty-three consecutive HCM patients who were examined both by LGE-CMR and by endomyocardial biopsy at Kanazawa University Hospital were retrospectively studied. HCM was diagnosed by the presence of a non-dilated and hypertrophied left ventricle on two-dimensional echocardiography (wall thickness ≥ 13 mm) in the absence of another disease that could account for the hypertrophy [17]. End-stage HCM was defined as a left ventricular ejection fraction <50% by two-dimensional echocardiography [12]. Two patients were excluded from the study due to unsatisfactory biopsy samples (i.e., samples yielding only endocardium). Thus, data from a total of 21 patients were included in the analyses of this study. Written informed consent was obtained from every patient before respective procedure (LGE-CMR or endomyocardial biopsy). The study complies with the Declaration of Helsinki. The study protocol was approved by the Bioethical Committee on Medical Research, School of Medicine, Kanazawa University.

### Electrocardiography

A standard 12-lead ECG (0.5 to 150 Hz, 25 mm/sec, 10 mm/mV) was recorded in the supine position during quiet respiration. Pathological Q waves were defined as follows; Q wave >1/3 of the ensuing R wave in depth and/or >40 msec in duration in at least 2 contiguous leads except aVR [18]. Negative T waves >10 mm in depth in any leads were defined as giant negative T waves [18]. Absence of Q waves in leads I, aVL, V5 and V6 was defined as absent Q waves in lateral leads [18].

### Echocardiography

Standard M-mode and two-dimensional echocardiographic studies were performed to identify and quantify morphological features of the left ventricle in accordance with the guideline of the American Society of Echocardiography [19]–[20].

### Endomyocardial biopsy and histological assessments of fibrosis

**Figure 1:**
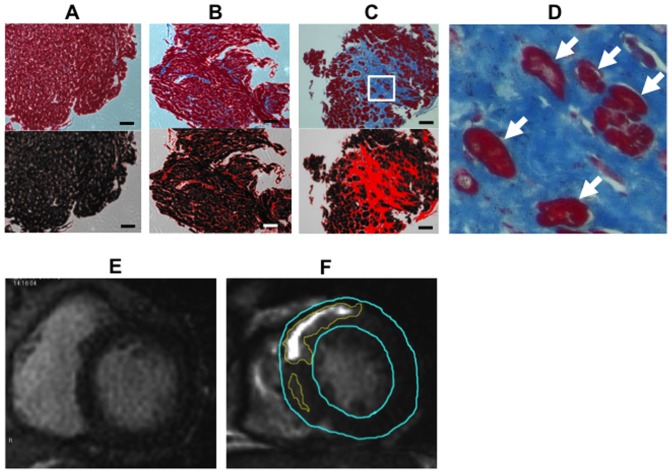
Qualitative and quantitative assessments of myocardial fibrosis in endomyocardial biopsy and LGE-CMR in the HCM patients. (**A**–**C**) Typical histology from endomyocardial biopsy. Upper panels; no pathological fibrosis (**A**), increased interstitial fibrosis (**B**), and severe myocardial scarring (**C**). Slides were stained with Azan dye. Interstitial fibrosis was defined as increased interstitial collagen without evidence of cardiomyocyte loss [9], [22]. Lower panels: areas of fibrosis visualized by ImageJ software. Fibrosis volume fraction determined by digital planimetry was 2% (**A**), 15% (**B**) and 45% (**C**), respectively. Myocardial scarring was defined as increased interstitial collagen with evidence of cardiomyocyte loss [9], [22]. The presence of islands of surviving cardiomyocyte among fibrotic tissues was considers as evidence of cardiomyocyte loss [23]. (**D**) Islands of surviving cardiomyocyte (arrows) among fibrotic tissues observed with high magnification of Image C. (**E and F**) Defining and quantifying LGE. Representative HCM patients with no LGE (**E**) and with positive LGE (**F**). Areas of LGE (traced by yellow lines) were quantified by manual planimetry and presented as percentage of left ventricular areas (traced by blue lines). Total of 6 short axes views were analyzed in each HCM patient. The LGE volume fraction was 0% in Case D, and 16% in Case E. Scale bars represent 200 µm. CMR  =  cardiac magnetic resonance imaging, HCM  =  hypertrophic cardiomyopathy, LGE  =  late gadolinium enhancement.

Selection of patients for endomyocardial biopsy was clinically determined according to a scientific statement regarding the role of endomyocardial biopsy in the management of cardiovascular disease [7]. Myocardial biopsy samples were taken from the right ventricular septal wall using a flexible bioptome [21]. No major complications such as cardiac rupture, pericardial tamponade or complete atrioventricular block occurred during endomyocardial biopsy. Sections were stained with hematoxylin-eosin and Azan dye and evaluated to identify interstitial and myocardial scarring. Interstitial fibrosis was defined as increased interstitial collagen without evidence of cardiomyocyte loss [9], [22]. Myocardial scarring was defined as increased interstitial collagen with evidence of cardiomyocyte loss [9], [22]. The presence of islands of surviving cardiomyocyte among fibrotic tissues was considers as evidence of cardiomyocyte loss [23]. Representative histopathological images of interstitial fibrosis and myocardial scarring were shown in Figures 1B and 1C–D, respectively. The collagen volume fraction was manually quantified using ImageJ software by 2 experienced investigators (T.K., Y.N.) who were blinded to the clinical information of the patients. The percentage area of fibrosis in each section was obtained by dividing the sum of the fibrotic areas of the section by that of the total tissue area (Figures 1A–C) [24].

### CMR Acquisition

CMR imaging was performed with a 1.5 Tesla scanner (GE). Delayed-enhancement images for detection of fibrosis were obtained 10 minutes after intravenous administration of gadolinium–DTPA (0.2 mmol/kg) [25]. Total of 6 short axis images were analyzed to qualify and quantify LGE. LGE was defined by a signal intensity of > 6 standard deviations above normal myocardium [26]. The LGE area was measured by manual planimetry using ImageJ software (National Institutes of Health, Bethesda, Maryland) in each short-axis image [27]. The area of the left ventricle was determined by manually tracing epicardial and endocardial borders in each short-axis image. The percentage area of LGE was then calculated by dividing the sum of the LGE areas by that of the total left ventricular area [27]. Data were presented both as percentage of the left ventricular area and as a binomial variable; positive/negative (Figures 1E and F). CMR data were analyzed by 2 experienced investigators (E.M., Y.N.) with consensus without knowledge of the clinical information of the patients.

### Statistical Analysis

Values are expressed as the mean ± standard deviation. Correlation was assessed by linear regression analysis and the Pearson correlation coefficient. Unadjusted and multivariate-adjusted linear regression analyses were used to assess the associations between the clinical parameters and the microscopic collagen fraction. First, each parameter was univariately analyzed in the regression model using the percentage area of microscopic fibrosis as the dependent variable (unadjusted model). Then, significant variables in the unadjusted analysis were subjected to the multivariate analysis together with age and gender (Multivariate model). Sensitivity, specificity, positive predictive value (PPV), and negative predictive value (NPV) were defined as previously reported [28]. P <0.05 was considered statistically significant.

## Results

### Patients Characteristics

Baseline characteristics of the 21 patients with HCM are summarized in Table 1. Of these 21 patients, 14 (67%) had asymmetric septal hypertrophy, and 3 (15%) had the phenotype of end-stage HCM with decreased left ventricular ejection fraction (<50%). The average maximal left ventricle wall thickness was 17.9 ± 5.6 mm. Pathological Q waves and giant negative T waves were observed in 2 (14%) and 5 (24%) of the 21 patients, respectively.

**Table 1:** Characteristics of the 21 patients with HCM.

	N = 21
Age (years)	57.8 ± 11.1
Male/Female, n	16 & 5
Phenotype	
ASH, n (%)	14 (67)
Apical, n (%)	1 (5)
Diffuse, n (%)	3 (14)
End-stage HCM, n (%)	5 (24)
Echocardiogram	
MWT (mm)	17.9 ± 5.6
IVST (mm)	16.8 ± 6.0
PWT (mm)	11.1 ± 2.1
LVDD (mm)	50.0 ± 11.0
LVEF (%)	61.2 ± 19.2
LAD (mm)	45.6 ± 7.4
Electrocardiogram	
AF, n (%)	2 (9)
CRBBB, n (%)	4 (19)
CLBBB, n (%)	0 (0)
QRS axis (degree)	28.6 ± 52.4
QRS width (msec)	115.3 ± 20.2
QTc interval (msec)	440.6 ± 35.9
SV1 + RV5 (mV)	4.0 ± 2.1
Pathological Q waves, n (%)	3 (14)
Giant negative T waves, n (%)	5 (24)
Absent Q waves in lateral leads, n (%)	8 (38)

AF  =  atrial fibrillation; ASH  =  asymmetrical septal hypertrophy; CLBBB  =  complete left bundle branch block; CRBBB  =  complete right bundle branch block; HCM  =  hypertrophic cardiomyopathy; IVST  =  intraventricular septal wall thickness; LAD  =  left atrial dimension; LVDD  =  left ventricular end-diastolic dimension; LVEF  =  left ventricular ejection fraction; MWT  =  maximal wall thickness; PWT  =  posterior wall thickness.

### Qualitative and qualitative data of fibrosis by endomyocardial biopsy and LGE-CMR

**Figure 2:**
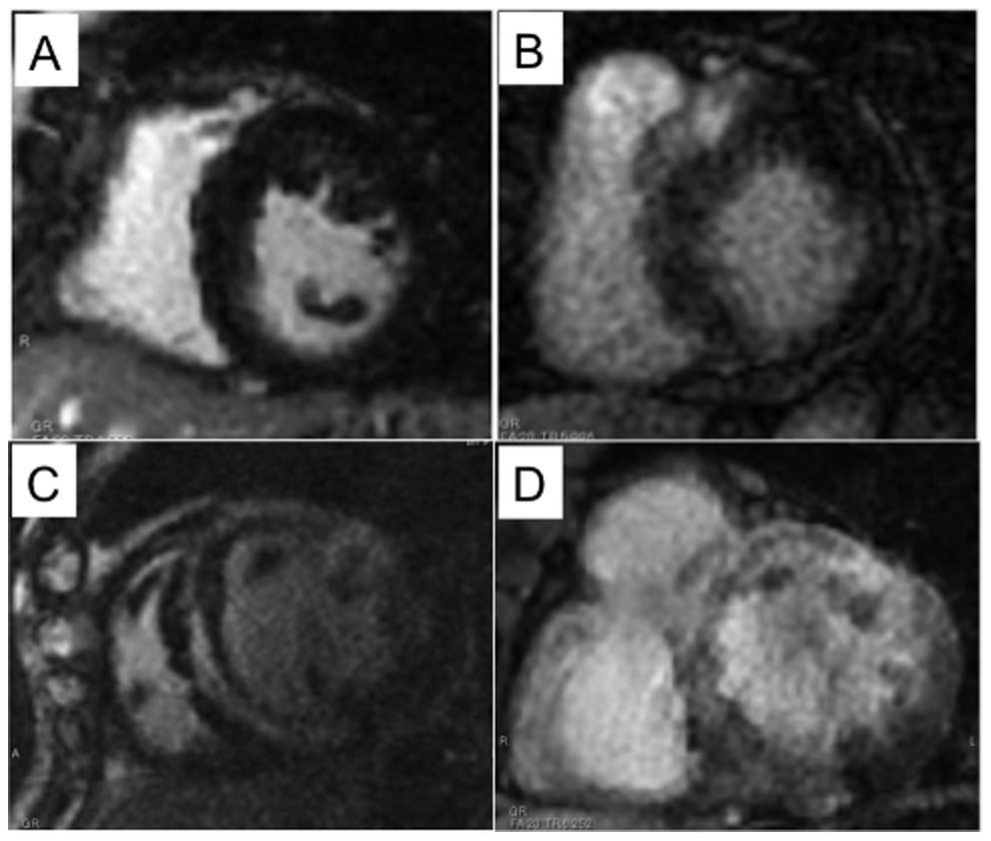
Representative patterns of LGE in the HCM patients. (**A**) No LGE. (**B**) Patchy midwall LGE in the anterior wall. (**C**) Linear midwall LGE in anteroseptal and lateral wall. (**D**) Extensive midwall and subendocardial LGE in the diffuse left ventricle.

Qualitative assessments of myocardial fibrosis in biopsy specimens revealed that interstitial fibrosis without myocardial scarring was present in 11 (52%) of the 21 patients, and interstitial fibrosis with myocardial scarring was present in 6 (29%) of them (Table 2). The mean collagen volume fraction in myocardial biopsy samples was 19.0 ± 16.8%. The extent of fibrosis varied from no pathological fibrosis to severe myocardial scarring (Figures 1 A–C). LGE was present in 17 (81%) of the 21 patients with HCM and was predominantly located in the anteroseptal segments (Table 3). The mean extent of LGE in the left ventricle was 8.5 ± 9.7%. The patchy midwall pattern, linear midwall pattern, and subendocardial pattern of LGE were observed in 17 (81%), 9 (43%), and 3 (14%) of the 21 HCM patients, respectively (Table 3). Representative images of various patterns of LGE were demonstrated in Figure 2.

**Table 2:** Histological characteristics of fibrosis in biopsy specimens in the 21 patients with HCM.

	N = 21
Fibrosis present, n (%)	17 (81)
Types of fibrosis, n (%)	
Interstitial only	11 (52)
Scarring only	0 (0)
Interstitial and scarring	6 (29)
Total fibrosis fraction (%)	19.0 ± 16.8

Interstitial fibrosis; increased interstitial collagen without evidence of myocyte loss. Myocardial scarring; increased interstitial collagen with evidence of myocyte loss [9], [22]. The presence of islands of surviving cardiomyocyte among fibrotic tissues was considers as evidence of cardiomyocyte loss [23]. HCM as in Table 1.

**Table 3:** Characteristics of LGE-CMR in the 21 patients with HCM.

	N = 21
LGE present, n (%)	17 (81)
Location of LGE, n (%)	
Anterior	12 (57)
Septal	15 (71)
Inferior	10 (47)
Lateral	6 (29)
Posterior	3 (14)
Apex	5 (24)
Patterns of LGE, n (%)	
Patchy midwall	17 (81)
Linear midwall	9 (43)
Subendocardial	3 (14)
Epicardial	0 (0)
Transmural	0 (0)
LGE volume fraction (%)	8.5 ± 9.7

CMR  =  cardiac magnetic resonance imaging; LGE  =  late gadolinium enhancement. HCM as in Table 1.

### Diagnostic values of LGE-CMR for predicting microscopic myocardial scarring in biopsied specimens in HCM

We first investigated whether LGE in the ventricular septum predicted microscopic myocardial scarring in small biopsied specimens in the 21 HCM patients. For predicting microscopic myocardial scarring, LGE showed an excellent sensitivity (100%); all of the 6 patients who had microscopic myocardial scarring showed LGE in the ventricular septum. In contrast, 6 (40%) of the 15 patients who did not have microscopic myocardial scarring were negative for LGE in the ventricular septum. Thus, the sensitivity, specificity, PPV, NPV and accuracy of LGE in the ventricular septum for predicting microscopic myocardial scarring were 100%, 40%, 40%, 100% and 57%, respectively (Table 4). We then investigated the diagnostic values of LGE in the whole heart for predicting microscopic myocardial scarring in biopsied specimens in the 21 HCM patients. The sensitivity of LGE in the whole heart remained 100%, but the specificity decreased (27%). The sensitivity, specificity, PPV, NPV and accuracy were 100%, 27%, 35%, 100% and 48%, respectively (Table 4).

**Table 4:** Diagnostic values of LGE for detecting microscopic myocardial scarring or interstitial fibrosis in biopsied specimens in the HCM patients (N  =  21).

	Sensitivity	Specificity	PPV	NPV	Accuracy
Detecting microscopic myocardial scarring					
LGE in ventricular septum	100%	40%	40%	100%	57%
LGE in whole heart	100%	27%	35%	100%	48%
Detecting microscopic interstitial fibrosis					
LGE in ventricular septum	76%	50%	87%	33%	29%
LGE in whole heart	88%	50%	88%	50%	19%

NPV  =  negative predictive value; PPV  =  positive predictive value. HCM as in Table 1.

LGE in the ventricular septum showed poor diagnostic accuracy for predicting microscopic interstitial fibrosis in the 21 HCM patients; the sensitivity, specificity, PPV, NPV and accuracy were 76%, 50%, 87%, 33% and 29%, respectively (Table 4). Additionally, the sensitivity, specificity, PPV, NPV and accuracy of LGE in the whole heart for predicting microscopic interstitial fibrosis were 88%, 50%, 88%, 50% and 19%, respectively (Table 4).

The study population included 5 patients with end-stage HCM (Table 1). Even when these 5 end-stage HCM patients were excluded (n  =  16), LGE showed similar diagnostic values for detecting microscopic myocardial scarring or interstitial fibrosis (Table 5); the sensitivity, specificity, PPV, NPV and accuracy of LGE in the whole heart for predicting microscopic myocardial scarring were 100%, 33%, 33%, 100% and 50%, respectively (Table 5). Additionally, the sensitivity, specificity, PPV, NPV and accuracy of LGE in the whole heart for predicting microscopic interstitial fibrosis were 85%, 67%, 92%, 50% and 19%, respectively in the 16 HCM patients (Table 5).

**Table 5:** Diagnostic values of LGE for detecting microscopic myocardial scarring or interstitial fibrosis in biopsied specimens in the HCM patients with preserved systolic function (N  =  16).

	Sensitivity	Specificity	PPV	NPV	Accuracy
Detecting microscopic myocardial scarring					
LGE in ventricular septum	100%	50%	40%	100%	63%
LGE in whole heart	100%	33%	33%	100%	50%
Detecting microscopic interstitial fibrosis					
LGE in ventricular septum	69%	67%	90%	33%	31%
LGE in whole heart	85%	67%	92%	50%	19%

HCM as in Table 1. NPV and PPV as in Table 4.

### Relationships between the LGE fraction and the microscopic collagen fraction

**Figure 3:**
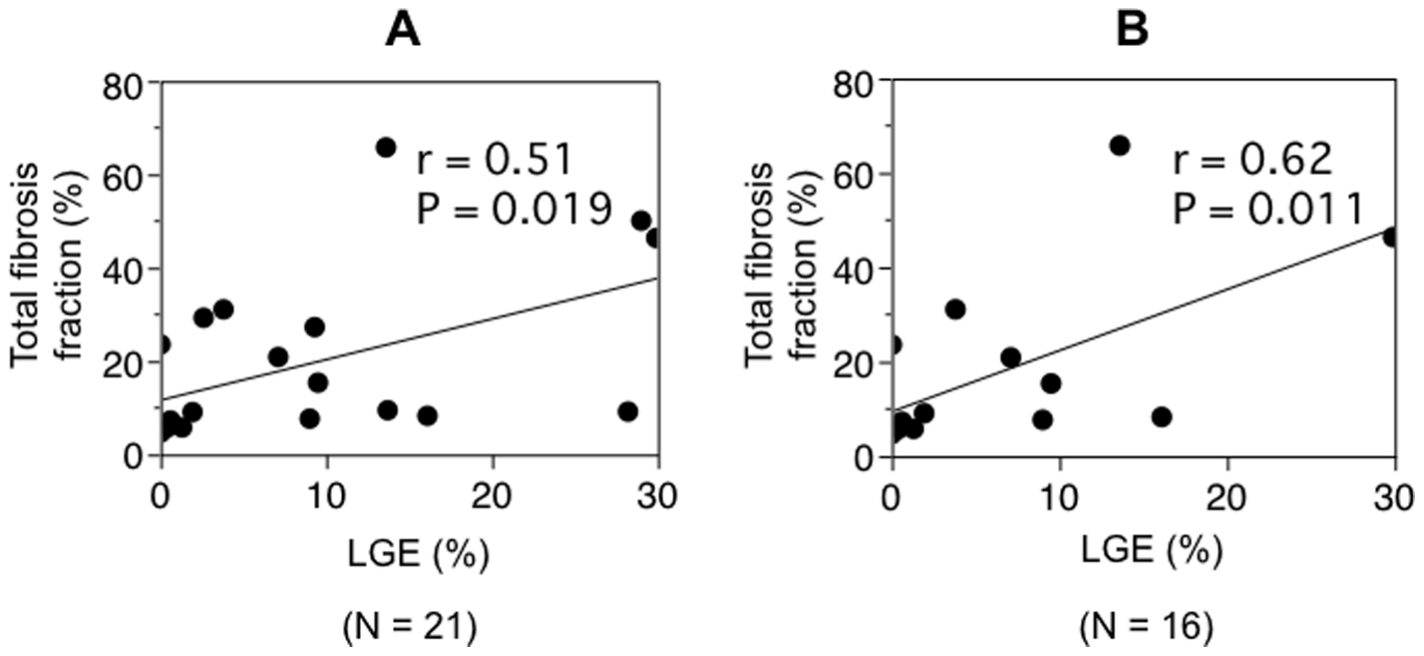
Relationships between the microscopic fibrosis fraction and the LGE fraction in the HCM patients. (**A**) A fair correlation was found between the LGE fraction and the total fibrosis fraction in biopsied specimens (N  =  21). (**B**) To examine whether the LGE fraction correlated with the microscopic collagen fraction in HCM with preserved LV systolic function, 5 end-stage HCM patients were excluded from analyses. In the remaining 16 HCM patients, a correlation between the LGE fraction in the whole heart and the microscopic collagen fraction in biopsied specimens was observed (r  =  0.62, p  =  0.011). Abbreviations as in Figure 1.

Since the results showed an excellent sensitivity of LGE in the whole heart for predicting microscopic myocardial scarring in biopsied specimens, we then asked whether or not the extent of LGE in the whole heart predicted the microscopic collagen fraction. Unadjusted regression analyses demonstrated a correlation between the LGE fraction in the whole heart and the microscopic collagen fraction in biopsied specimens (r  =  0.51, p  =  0.019) (Figure 3A, Table 6). Multivariate adjusted regression analyses revealed that the extent of LGE in the whole heart was the independent variable related to the microscopic collagen fraction in biopsied specimens, even after adjustments for clinical cofounders (Table 6).

**Table 6:** Predictors of the collagen fraction in biopsied specimens (unadjusted and multivariate-adjusted regression analyses).

	Univariate			Multivariate		
	β	95% CI	P value	β	95% CI	P value
% LGE	0.51	0.092 – 0.92	0.019	0.59	0.15 – 1.0	0.012
QRS width	0.47	0.045 – 0.89	0.032	0.016	−0.46 – 0.49	0.94
AF	−0.42	−0.85 – 0.018	0.059			
Pathological Q	−0.40	−0.84 – 0.046	0.076			
QTc interval	0.25	−0.22 – 0.71	0.27			
LVEF	−0.24	−0.71 – 0.23	0.29			
Gender	−0.21	−0.26 – 0.67	0.37	0.5	−0.018 – 1.0	0.06
SV1 + RV5	−0.20	−0.52 – 0.24	0.45			
Age	−0.1	−0.58 – 0.38	0.66	−0.31	−0.78 – 0.16	0.18
QRS axis	−0.045	−0.52 – 0.44	0.85			
LVDD	0.04	−0.44 – 0.52	0.86			
Absent Q in lateral leads	0.039	−0.44 – 0.52	0.87			
MWT	−0.019	−0.50 – 0.46	0.93			
PWT	0.015	−0.47 – 0.50	0.94			

CI  =  confident interval. AF, EF, HCM, LGE, LVDD, MWT and PWT as in Tables 1 and 3.

We next examined whether the LGE fraction correlated with the microscopic collagen fraction even when the 5 end-stage HCM patients were excluded from analyses. In the remaining 16 HCM patients with preserved left ventricular ejection fraction, a correlation between the LGE fraction in the whole heart and the microscopic collagen fraction in biopsied specimens was observed (r  =  0.62, p  =  0.011) (Figure 3B).

## Discussion

These data demonstrate that the sensitivity of LGE was excellent for prediction of microscopic myocardial scarring in HCM although there was a compromise with specificity. Furthermore, the extent of LGE independently correlated with the severity of microscopic collagen fraction in biopsied specimens in HCM patients.

### Diagnostic values of LGE for predicting microscopic myocardial scarring in biopsied specimens in HCM

**Figure 4:**
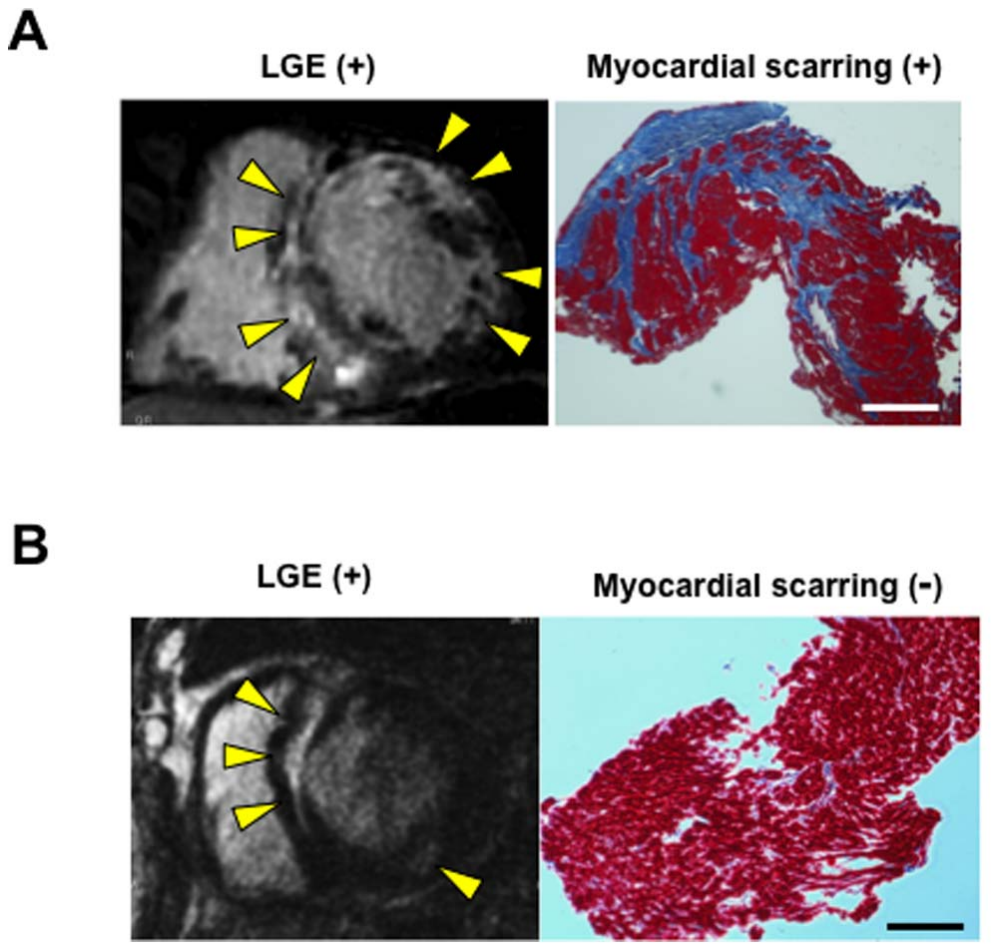
Concordant and discordant appearance of fibrosis in LGE-CMR and endomyocardial biopsy in the HCM patients. (**A**) Myocardial scarring was observed both in CMR and endomyocardial biopsy. (**B**) LGE was present in CMR, but there was no evidence of microscopic myocardial scarring. Scale bars represent 200 µm. Abbreviations as in Figure 1.

There is excellent agreement between the location of myocardial scarring determined postmortem and the antemortem LGE location on CMR in HCM [13]. However, it has been unknown whether LGE predicts microscopic myocardial scarring in small biopsied specimens in HCM. In this study, we demonstrated that LGE had an excellent sensitivity (100%) for predicting microscopic myocardial scarring in biopsied specimens both in the ventricular septum and in the whole heart (Table 4). Thus, all of the HCM patients with microscopic myocardial scarring showed LGE, as shown in a representative case in Figure 4A. Despite the excellent sensitivity, there was a compromise with the specificity. LGE was present in most (73%) of the HCM patients who did not show microscopic myocardial scarring, as shown in Figure 4B. The patchy nature of fibrosis in HCM [9]–[13] might contribute to the low specificity of LGE for predicting microscopic myocardial scarring in HCM. Furthermore, some controversies exist over the correlations between LGE and myocardial fibrosis in biopsied specimens, which could in part be explained by different biopsy procedures and the specimen size in each study [6], [8], [29]–[31]. Nonetheless, given the excellent sensitivity and NPV of LGE for predicting microscopic myocardial scarring, LGE-CMR may play an important role in preventing unnecessary and invasive endomyocardial biopsies in the clinical assessment of HCM.

Although recent data showed that the LGE fraction at the myectomy site accurately reflected the histopathological fibrosis fraction in large myectomy specimens from HCM patients [32], it has been unclear whether the LGE fraction in the whole HCM heart correlates with the fibrosis fraction in small biopsied specimens. Our data demonstrated that the extent of LGE in the whole heart could reflect the severity of microscopic collagen fraction in HCM (Figure 3, Table 6). Together, these findings indicate that qualitative and quantitative assessments of noninvasive LGE-CMR may be used to predict both the presence and the severity of microscopic myocardial scarring in HCM. Because myocardial scarring in the HCM heart has been associated with heart failure, malignant ventricular arrhythmias and sudden cardiac death [14]–[16], further studies are needed to clarify whether LGE has different ability to predict the occurrence of cardiac events compared to microscopic myocardial scarring in HCM patients.

### Limitations

One limitation of this study was the small sample size. Another limitation is a selection bias regarding the severity of the disease because this study population was enrolled in a tertiary referral center. Specifically, a high prevalence (24%) of end-stage HCM patients in the studied population might have affected the results. Although correlations between LGE and microscopic fibrosis were observed even when these end-stage HCM patients where excluded from the analyses (Figure 3B, Table 5), our results may not be applicable to all HCM patients.

### Conclusions

Although there was a compromise in specificity, LGE showed an excellent sensitivity for predicting microscopic myocardial scarring in small biopsied specimens. Moreover, the quantitative LGE fraction in the whole heart correlated with the severity of microscopic collagen fraction in biopsied specimens in HCM. These findings suggest that qualitative and quantitative assessments of noninvasive LGE-CMR may be used to predict both the presence and the severity of microscopic myocardial scarring in HCM.
